# Successful management of bleeding from gastric fundic artery malformation using endoscopic intensive ligation combined with clips

**DOI:** 10.1055/a-2607-8315

**Published:** 2025-07-01

**Authors:** Jing Ding, Ke Zhu, Wei Zhang, Jing Jin, Chao Ma

**Affiliations:** 1 Department of Gastroenterology, No. 2 People’s Hospital of Fuyang City, Fuyang, China; 2 Department of Gastroenterology, Shimenkan Community Health Service Center, Nanjing, China

A 69-year-old man was hospitalized with hematochezia for 10 days. A previous gastroscopy had revealed gastric varices. However, a computed tomography (CT) scan showed an abnormal artery originating from the abdominal aorta and penetrating the gastric wall, forming tortuous vessel clusters in the gastric fundus. Gastroscopy revealed a red ulcer on the vessel cluster surface, which was considered the site of bleeding. The vessel blood flow was identified to be arterial by endoscopic ultrasound ( Fig. 1 ).

**Fig. 1:**
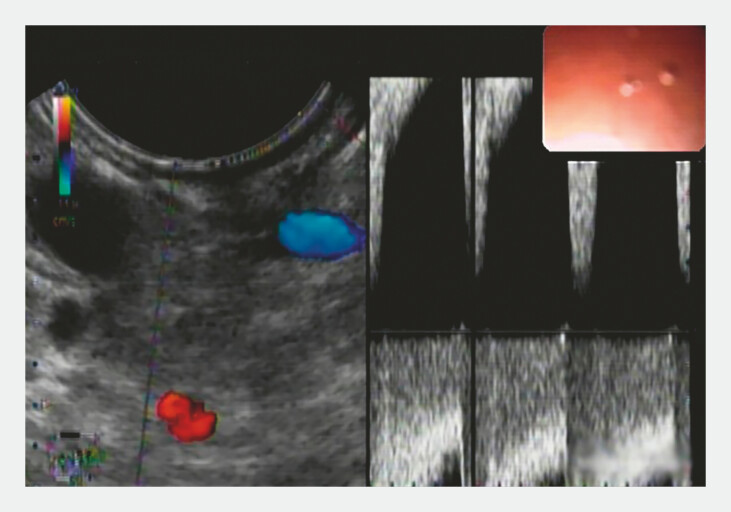
The vessel blood flow was identified to be arterial by endoscopic ultrasound.

A clip (Resolution Clip; 235 cm × 28 mm; Boston Scientific, Marlborough, Massachusetts, USA) was first clamped at the upstream artery of the ulcerated surface to restrict blood flow to the ulcer, and then a band (Speedband Superview; 7 Bands; Boston Scientific) was used to ligate the ulcer. The clustered tortuous arteries were subsequently banded with eight bands intensively. Three clips were used to secure the roots of the ligature bands to prevent bleeding in the event of the untimely shedding of the ligature bands ( Fig. 2 , Video 1 ).

**Fig. 2:**
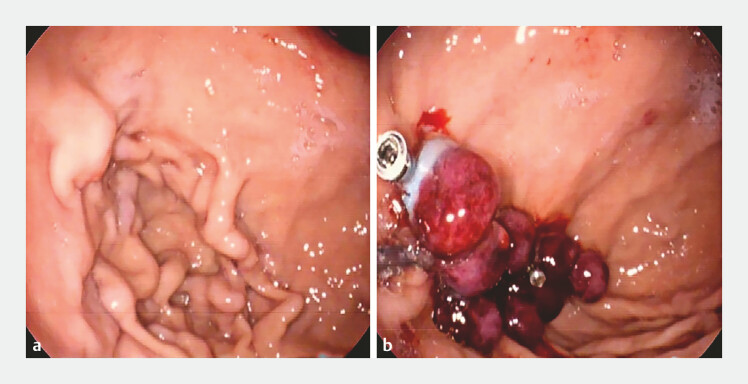
Endoscopy images before and after treatment. **a** Gastric fundic artery malformation may mimic gastric varices. **b** Endoscopic intensive ligation combined with clips was used to treat the artery malformation; a total of nine bands and four clips was used.

Management of bleeding from gastric fundic artery malformation using endoscopic intensive ligation combined with clips.Video 1

The next day, the patient experienced fever and abdominal pain, and CT showed splenic infarction. A comparative analysis of two CT scans and vascular images showed that the blood supply to the middle part of the spleen was predominantly derived from the cluster of tortuous arteries ( Fig. 3 ).

**Fig. 3:**
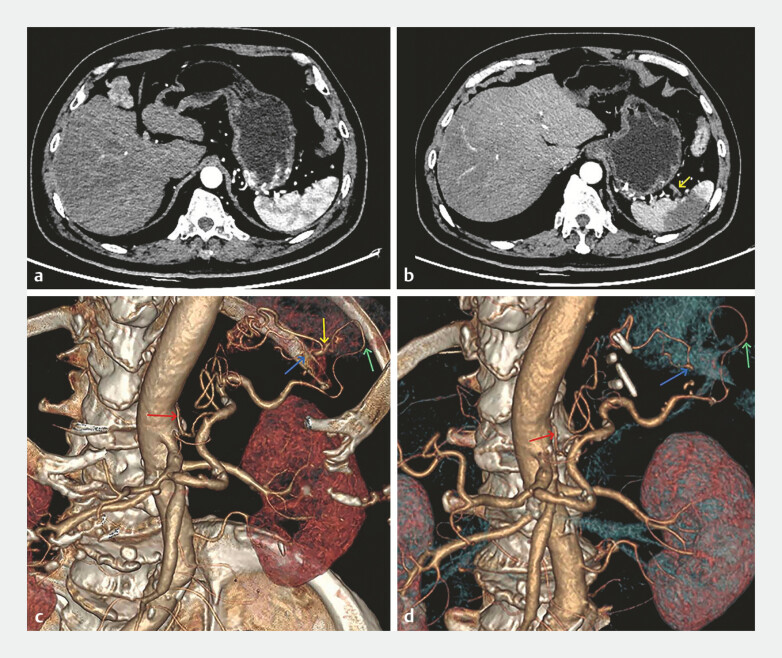
Computed tomography scans ( **a, b** ) and vascular imaging ( **c, d** ) before and after treatment. **a, c** An abnormal artery (red arrow) originating from the abdominal aorta penetrated the gastric wall and formed tortuous vessel clusters in the gastric fundus. The arterial cluster then branched out and supplied the upper and middle parts of the spleen (yellow and blue arrows). The lower part of the spleen was supplied by the splenic artery (green arrow) directly from the abdominal aorta. **b, d** After endoscopic treatment, the blood supply to the middle part of the spleen (yellow arrow) was restricted.

The 1-month post-treatment gastroscopy revealed post-treatment scars and residual clips, and confirmed that most of the clustered tortuous arteries had disappeared. The CT scan showed improvement in splenic infarction ( Fig. 4 ). At the 6-month follow-up, the patient did not report any symptoms and no bleeding was observed.

**Fig. 4:**
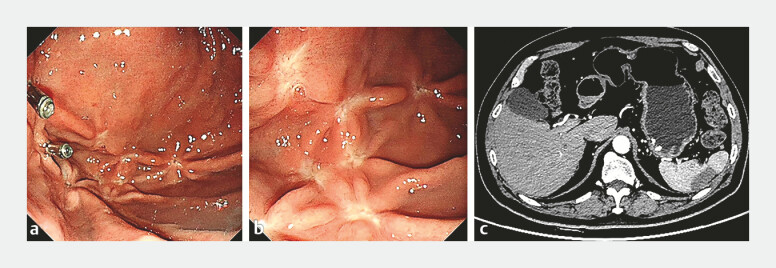
Gastroscopy and computed tomography (CT) scan a month after endoscopic treatment. **a, b** Gastroscopy showed the post-treatment scars and residual clips, and confirmed that most of the clustered tortuous arteries had disappeared. **c** The CT scan showed improvement in splenic infarction.

Gastric fundic artery malformation may mimic gastric varices, and inappropriate treatment can lead to serious consequences 1 . To our knowledge, this is the first report of a novel technique for gastric fundic artery malformation, which provides a new option for similar cases.

Endoscopy_UCTN_Code_TTT_1AO_2AD

## Notes

Endoscopy E-Videos https://eref.thieme.de/e-videos
*E-Videos* is an open access online section of the journal *Endoscopy* , reporting on interesting cases and new techniques in gastroenterological endoscopy. All papers include a high-quality video and are published with a Creative Commons CC-BY license. Endoscopy E-Videos qualify for HINARI discounts and waivers and eligibility is automatically checked during the submission process. We grant 100% waivers to articles whose corresponding authors are based in Group A countries and 50% waivers to those who are based in Group B countries as classified by Research4Life (see: https://www.research4life.org/access/eligibility/ ). This section has its own submission website at https://mc.manuscriptcentral.com/e-videos .
